# Non-deforestation drivers of fires are increasingly important sources of aerosol and carbon dioxide emissions across Amazonia

**DOI:** 10.1038/s41598-019-53112-6

**Published:** 2019-11-18

**Authors:** William T. Morgan, Eoghan Darbyshire, Dominick V. Spracklen, Paulo Artaxo, Hugh Coe

**Affiliations:** 1 School of Earth & Environmental Sciences, University of Manchester, Manchester, UK; 2 School of Earth and Environment, University of Leeds, Leeds, UK; 3 Physics Institute, University of Sao Paulo, Sao Paulo, Brazil

**Keywords:** Atmospheric science, Environmental impact

## Abstract

Deforestation rates have declined substantially across the Brazilian Legal Amazon (BLA) over the period from 2000–2017. However, reductions in fire, aerosol and carbon dioxide have been far less significant than deforestation, even when accounting for inter-annual variability in precipitation. Our observations and analysis support a decoupling between fire and deforestation that has exacerbated forest degradation in the BLA. Basing aerosol and carbon dioxide emissions on deforestation rates, without accounting for forest degradation will bias these important climate and ecosystem-health parameters low, both now and in the future. Recent increases in deforestation rate since 2014 will enhance such degradation, particularly during drought-conditions, increasing emissions of aerosol and greenhouse gases. Given Brazil’s committed Nationally Determined Contribution under the Paris Agreement, failure to account for forest degradation fires will paint a false picture of prior progress and potentially have profound implications for both regional and global climate.

## Introduction

Amazonia is one of the world’s most important tropical regions and is suffering substantial pressures from human occupation, expansion of agricultural activities and climate change. The biological functioning of the forest is being altered by smoke from biomass burning, changes in precipitation patterns and extreme events such as frequent intense droughts^1^. The majority of Amazonia is located in Brazil, demarcated as the Brazilian Legal Amazon (BLA). Tropical moist broadleaf forest covers 81% of the area, with tropical grasslands, savannah and shrublands composing 16%^2^. The majority of BLA states are dominated by tropical forests, with Cerrado (savannah-like areas) also present in significant portions of Mato Grosso, Tocantins and Maranhão.

Large-scale deforestation of the BLA to clear vegetation and maintain agricultural lands has been recorded as a regular annual occurrence since the 1970s. As of 2018, approximately 800,000 $k{m}^{2}$ of the BLA has been deforested^3,4^, which represents close to 20% of the original area of the Amazon rainforest in the BLA. Since 2004, official figures for the BLA produced by the Brazilian National Institute for Space Research^4^ have shown a significant decline in annual deforestation^5–7^. International and national policy interventions have been identified as being major drivers of the observed reductions^8–11^. The primary means of clearing an area is through so-called ‘slash-and-burn’ agriculture, where the forest is cut and subsequently burned^12^. The cutting and burning stages are usually separated as the fallen wood is given time to dry due to very high moisture. Deforestation peaks in June/July, approximately three months after the peak in the rainy season, while biomass burning peaks in September when fire counts reach their maximum^13^. The annual burning season typically runs from August to October, with the associated fires producing large amounts of gas and particulate phase material that have a range of environmental and health consequences.

Biomass burning aerosol is a global phenomenon that represents a significant uncertainty in assessments of the impact of atmospheric aerosol on the Earth System. Biomass burning aerosol impacts global climate and the hydrological cycle in tropical and savannah environments, as well as boreal regions. Biomass burning aerosol-radiation interactions are very poorly constrained with the global radiative forcing central estimate spanning positive and negative values due the uncertain balance between scattering organic aerosols and absorbing black carbon^14^. The impact of the aerosol is most keenly felt on regional and seasonal time scales where significant radiative perturbations may occur, particularly over South America, Southern Africa and Southeast Asia. The overlap between emissions and the abundance of water vapour in the tropics has major implications and potential for significant aerosol-cloud interactions. In the BLA, the smoke produced during the burning season builds up to form a large atmospheric aerosol burden^15^ that has been shown to affect climate^16^, visibility^17^, weather^18^, human health^19^ and the regional ecosystem^20,21^. Absorption of radiation by biomass burning smoke over the Amazon can inhibit cloud formation or even induce so-called cloud “burn-off”^22,23^. Additionally, biomass burning is a significant source of short lived climate forcers such as ozone, with changes in emissions of volatile organic compounds being important for atmospheric chemistry and organic aerosol formation.

As well as aerosol, biomass burning produces substantial emissions of long-lived greenhouse gases, particularly carbon dioxide and methane. From 2007–2016, global land-use change emissions, of which deforestation is a major component, contributed 1.3 $\pm$ 0.7 $GtCy{r}^{-1}$ compared to 9.4 $\pm$ 0.4 $GtCy{r}^{-1}$ from fossil fuels and industry^24^. According to figures submitted to the United Nations Framework Convention on Climate Change (UNFCCC), the Land Use, Land-Use Change and Forestry sector accounted for 78–80% of Brazil’s annual carbon dioxide emissions from 1990–2005, before falling to 42% in 2010^25^.

Deforestation rates have declined from 2004 onwards^26,27^ but total fire counts and burned area have exhibited a much slower decline^7,11,27–29^. Altered fire dynamics driven by deforestation and forest degradation have been identified as significant contributing factors to these observed trends. Feedbacks in the fire dynamics of closed canopy tropical forests increase future fire susceptibility, fuel loading and fire intensity, leading to accidental fires potentially causing more deforestation than intentional clearing in some regions^30^. From 1998–2006, fire occurrence actually increased in 59% of the area that experienced reduced deforestation rate^28^. This was ascribed to slashing and burning of secondary forests in already deforested areas that are not monitored by the official Instituto Nacional de Pesquisas Espaciais (INPE) deforestation assessments, as well as the continuous enlargement of forest edges and the increasing area of secondary forest cover that is more susceptible to fire. Such feedbacks have resulted in many fires in the Amazon no longer originating from deforestation itself, but from managed agricultural lands and those that escape from these managed lands^29^. Prior to the establishment of the Action Plan for Prevention and Control of Deforestation in Amazonia (PPCDAm) in 2004, deforestation explained 84% of active fire detections^13^. Once the PPCDAm was implemented, this fell to only 47% over the period from 2004–2015^7^. From 2003–2015, there has been a significant positive trend in the number of active fire counts per square kilometre deforested, with peaks in active fire counts more associated with extreme drought events than deforestation^7,27^. The most significant increases were in areas with only limited deforestation, suggesting that fires associated with forest degradation are becoming increasingly important^27^.

The other major controlling factor on trends in fire in the BLA is decreased precipitation, especially drought conditions, which exacerbate the factors associated with fire dynamics. For a one standard deviation decrease in precipitation, fire events and burned area increase by 11–15% and 18–27% respectively^11^. During the severe drought in 1998 associated with the $ElNi{\tilde{n}}o$-Southern Oscillation, the area of forest burned by understory forest fire (where below-canopy fire is the dominant type) was 13 times greater than the area burned during a year with average rainfall and twice the extent of the annual deforestation^31^. In 1995, a non-$ElNi{\tilde{n}}o$ year, understory fires were concentrated in transitional forests, while dense forest was the most affected during the $ElNi{\tilde{n}}o$ year^31^. During the 2005 drought, fire counts were 33% greater compared to the 1999–2005 mean and in the region most severely affected by the drought (the state of Acre), the area of leakage forest fires was more than five times greater than the area directly deforested^32^. Drought has been shown to significantly increase the number of fires in the region, even with decreased deforestation rates^13^. Continuing slash-and-burn deforestation procedures and the use of fires for land management will intensify this trend, as larger portions of forest edges become more exposed to the risk of fires^13^. Furthermore, the role of terrestrial water storage at the end of the wet season is an important control on fire activity in the following dry season^27^.

Previous work^5,15,33^ has established that the magnitude of the regional aerosol burden is strongly controlled by the intensity of the biomass burning season. However, trends in the aerosol burden and associated properties over the recent period of steep deforestation decline and de-coupling between deforestation and fire has received less scrutiny. Long-term satellite measurements of carbon monoxide (CO) have shown a negative trend that has been ascribed to falling deforestation rates, while also illustrating substantial increases in CO during drought years^34^. While the lifetimes of aerosol and CO differ, their common sources and regional build-up suggests similar trends will be observed for both. Satellite measurements of aerosol optical depth (AOD) over the period from 2001–2012 indicate enhanced AOD in drought years coupled with a negative trend overall, which was ascribed to declines in deforestation fires^19^. Carbon emissions in Amazonia are strongly associated with fire^7,35^ and are thus prone to the same changes in fire dynamics and processes outlined above.

This is the first study to investigate trends in aerosol and carbon dioxide emissions from the BLA and place them in the context of the observed changes in precipitation, deforestation, fire count and burned area. We focus on the past two decades of change in the BLA, finding a significant decoupling between fire and deforestation that exacerbates forest degradation and leads to lower reductions in aerosol and carbon dioxide emissions across the region than if deforestation was the primary driver of forest loss. Basing emissions of these important climate and ecosystem-health parameters on deforestation will bias them low, both now and in the future. Such erroneous estimates would paint a false picture of prior progress that feed through to Brazil’s commitments under the Paris Agreement Nationally Determined Contributions and potentially have profound implications for both regional and global climate.

## Results

Details on the data and methods used in the following section can be found later in the manuscript after the discussion section. This includes the calculation of precipitation-adjusted trends in fire parameters, AOD and carbon dioxide emissions, which aim to account for inter-annual variability in rainfall across the region to better understand the non-meteorological drivers of fire.

### Spatial distribution

**Figure 1:**
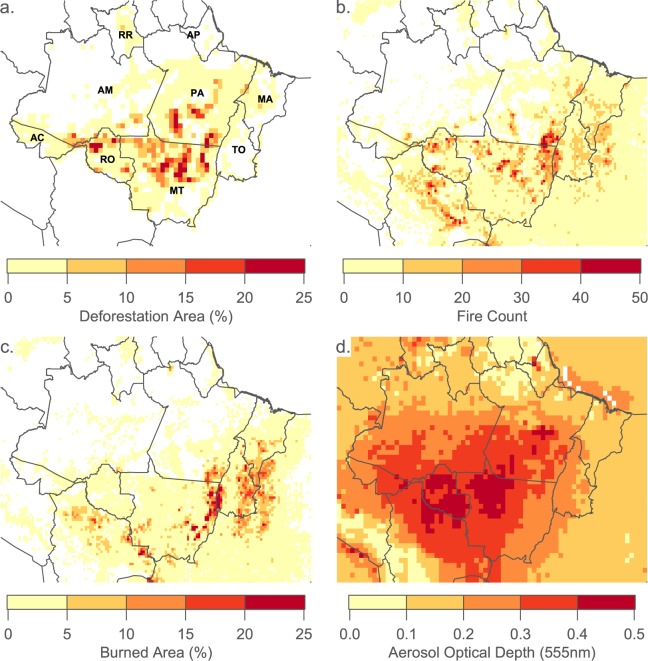
(**a**) Map of the Brazilian Legal Amazon (BLA) states and cumulative deforestation as a percentage of each 0.50${}^{\circ }$ pixel from May 2004 to July 2016 from INPE’s DETER data set. State names are as follows: AC - Acre, AM - Amazonas, RO - Rond${\hat{o}}$nia, RR - Roraima, MT - Mato Grosso, PA - Pará, AP - Amapá, TO - Tocantins and MA - Maranhão. (**b**) Map of average fire count on a 0.25$^{\circ }$ pixel grid from August 2001 to July 2016. (**c**) Map of average burned area from August 2001 to July 2016 as a percentage of each 0.25$^{\circ }$ pixel. (**d**) Map of Aerosol Optical Depth on a 0.50$^{\circ }$ pixel grid from August 2001 to July 2016. Fire count, burned area and aerosol optical depth is averaged over the main biomass burning months (August-October).

The spatial distribution of deforestation, fire, burned area and AOD is shown in Fig. 1. Fire, burned area and AOD is averaged over the main biomass burning months (August–October), while deforestation is the accumulated deforestation from May 2004 to May 2017.

Deforestation is primarily confined to the so-called ‘Arc of Deforestation’ encompassing northern Rond${\hat{o}}$nia and Mato Grosso, along with areas of central and southern Pará.

The spatial distribution of fire overlaps with deforestation in Rond${\hat{o}}$nia, Mato Grosso and south-eastern Pará. Fire is less prevalent in the central regions of Pará where significant deforestation occurs. Fires are also prevalent in eastern regions that are dominated by grassland and shrubland, rather than tropical forest environments.

Burned area is relatively low in the major deforestation areas and primarily overlaps with fire in the eastern regions (east-Mato Grosso, Tocantins and Maranhão). Burned area is greatest in Tocantins and southern Maranhão where the ecosystem is dominated by shrublands.

The maximum in the AOD spatial distribution is located over the central and western regions of the BLA relative to the major fire regions (driven by the predominant easterly air flow across the region^36^).

For the subsequent analysis, we will examine the observations for the BLA as a whole and for the major biomass burning states (Rond${\hat{o}}$nia, Mato Grosso, Pará and Tocantins), which are defined based on Fig. 1.

### Annual cycles

**Figure 2:**
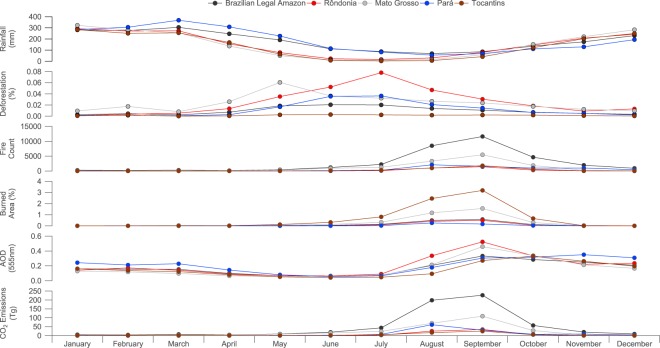
Monthly averages of rainfall, deforestation, fire count, burned area, Aerosol Optical Depth (AOD) and fire-related carbon dioxide emissions in the Brazilian Legal Amazon (BLA) and states where significant annual fire occurs (Rond${\hat{o}}$nia, Mato Grosso, Pará and Tocantins).

Figure 2 presents the annual cycle in rainfall, deforestation, fire, burned area and AOD across the BLA and major biomass burning states.

Within the BLA, precipitation has a broad peak from December-April, exceeding 200 $mm$ $mont{h}^{-1}$ on average. The peak in the wet season is reached in March, with over 300 $mm$ of rainfall on average. The major biomass burning states exhibit a similar cycle, with Pará generally receiving greater rainfall on average than the BLA as a whole during the peak in the wet season. Rond${\hat{o}}$nia, Mato Grosso and Tocantins experience similar rainfall on average compared to the whole BLA during the peak of the wet season, while being drier on average from April-to-July. We note that the transition from the wet-to-dry season varies regionally and inter-annually, which we explore in greater detail in the methods section.

Deforestation across the BLA as a whole has a fairly broad peak from May-July, but also with significant deforestation occurring through August and September. Peaks in deforestation are variable from region-to-region. Mato Grosso peaks in May, which is the earliest peak across the major biomass burning regions, with the deforestation rate extending through to October. Rond${\hat{o}}$nia and Pará exhibit a broad peak from May-October, with the maximum in July and June respectively. While deforestation is low over Tocantins, it exhibits a similarly broad peak, with maxima in June and September.

Fire count is greatest from August-October, with the peak in September across the BLA and the major biomass burning states, except for Pará, whose fire count in August is marginally greater than in September. The annual cycle in burned area is very similar to fire count across the BLA and the major biomass burning states. AOD increases from August onwards, peaking in September and remaining relatively enhanced until December. AOD in the wet season (January-April) has been linked to long-range transport of aerosols from Africa, bringing Saharan dust and African biomass burning to the northern part of the BLA^37^.

### Trends in deforestation rate, fire and aerosol

**Figure 3:**
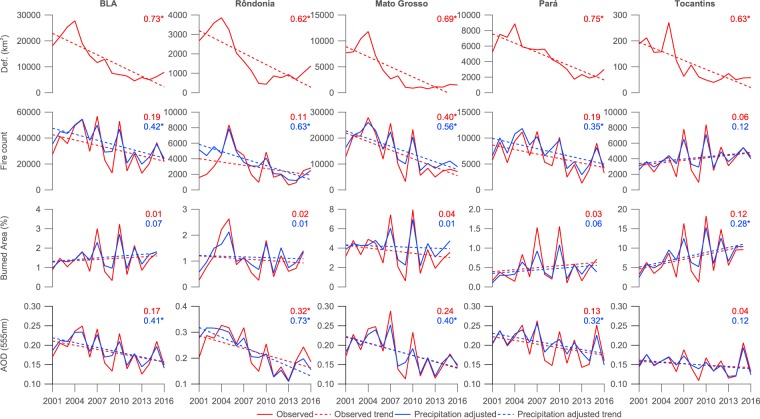
Trends in deforestation, fire and aerosol parameters from 2002–2016 centred on September in the Brazilian Legal Amazon and states where significant annual fire occurs (Rond${\hat{o}}$nia, Mato Grosso, Pará and Tocantins). Deforestation rates are from INPE’s PRODES data set. Coefficients of determination (${R}^{2}$) values are included for each sub-figure, where * signifies that the coefficient is significantly different from zero at the 95% confidence level.

Trends in deforestation, fire and aerosol parameters are shown in Fig. 3 plus Tables 1 and 2 for the BLA and the major biomass burning states. Statistically significant trends are based on the 95% confidence interval when applying a linear regression to the parameter of interest. Coefficients of determination (${R}^{2}$) between the parameter of interest and the year of the observation are also reported in Fig. 3 to indicate the strength of the relationship.

**Table 1:** Per annum trends in deforestation rates, fire counts, burned area, Aerosol Optical Depth (AOD) and total fire-related carbon dioxide emissions. Units are given in brackets. Errors are quoted as 95% confidence intervals around the linear regression slope. Values in bold are statistically significant at the 95% confidence level. Trends are shown for the full period and for the precipitation-adjusted (PA) time series.

Parameter	Data	BLA	Rond${\hat{o}}$nia	Mato Grosso	Pará	Tocantins
Deforestation (km${}^{2}$ year${}^{-1}$)	Full	$-{\bf{1376}}$	$-194$	$-{\bf{650}}$	$-{\bf{395}}$	$-{\bf{12}}$
		$\pm$**475**	$\pm$232	$\pm$**252**	$\pm$**131**	$\pm$**5**
Fire Count (# year${}^{-1}$)	Full	$-1366$	$-140$	$-{\bf{1100}}$	$-291$	107
		$\pm$1628	$\pm$232	$\pm$**768**	$\pm$343	$\pm$238
	PA	$-{\bf{1537}}$	$-{\bf{303}}$	$-{\bf{995}}$	$-{\bf{330}}$	95
		$\pm$**1025**	$\pm$**132**	$\pm$**503**	$\pm$**260**	$\pm$150
Burned Area (% year${}^{-1}$)	Full	0.022	$-0.020$	$-0.090$	0.017	0.42
		$\pm$0.11	$\pm$0.093	$\pm$0.27	$\pm$0.062	$\pm$0.66
	PA	0.030	$-0.0090$	$-0.028$	0.015	**0.42**
		$\pm$0.068	$\pm$0.063	$\pm$0.17	$\pm$0.035	$\pm$**0.40**
AOD (year${}^{-1}$)	Full	$-0.0036$	$-{\bf{0.0083}}$	$-0.0055$	$-0.0032$	$-0.0011$
		$\pm$0.0045	$\pm$**0.0069**	$\pm$0.0055	$\pm$0.0048	$\pm$0.0032
	PA	$-{\bf{0.0041}}$	$-{\bf{0.012}}$	$-{\bf{0.0052}}$	$-{\bf{0.0035}}$	$-0.0015$
		$\pm$**0.0029**	$\pm$**0.0043**	$\pm$**0.0037**	$\pm$**0.0029**	$\pm$0.0023
CO${}_{2}$ emissions (Tg year${}^{-1}$)	Full	$-11.6$	$-1.9$	$-13.0$	$-1.0$	2.6
		$\pm$38.0	$\pm$5.2	$\pm$17.1	$\pm$9.3	$\pm$4.8
	PA	$-14.5$	$-{\bf{5.5}}$	$-10.9$	$-0.8$	2.2
		$\pm$23.8	$\pm$**2.8**	$\pm$11.1	$\pm$6.6	$\pm$3.3

**Table 2:** Relative per annum percentage trends in deforestation rates, fire counts, burned area, Aerosol Optical Depth (AOD) and total fire-related carbon dioxide emissions in % year${}^{-1}$. Errors are quoted as 95% confidence intervals around the linear regression slope. Values in bold are statistically significant at the 95% confidence level. Trends are shown for the full period and for the precipitation-adjusted (PA) time series.

Parameter	Data	BLA	Rond${\hat{o}}$nia	Mato Grosso	Pará	Tocantins
Deforestation	Full	$-{\bf{7.58}}$	$-7.27$	$-{\bf{8.44}}$	$-{\bf{7.55}}$	$-{\bf{6.18}}$
		$\pm$**2.62**	$\pm$17.8	$\pm$**3.27**	$\pm$**2.49**	$\pm$**2.74**
Fire Count	Full	$-5.00$	$-8.85$	$-{\bf{8.59}}$	$-5.05$	3.46
		$\pm$5.96	$\pm$14.6	$\pm$**6.00**	$\pm$5.94	$\pm$7.71
	PA	$-{\bf{4.35}}$	$-{\bf{5.88}}$	$-{\bf{6.06}}$	$-{\bf{5.00}}$	3.69
		$\pm$**2.90**	$\pm$**2.56**	$\pm$**3.06**	$\pm$**3.94**	$\pm$5.81
Burned Area	Full	2.51	$-7.33$	$-2.85$	10.4	12.8
		$\pm$12.6	$\pm$34.8	$\pm$8.51	$\pm$38.1	$\pm$20.4
	PA	3.11	$-1.56$	$-0.728$	13.4	**17.1**
		$\pm$6.96	$\pm$10.9	$\pm$4.43	$\pm$31.9	$\pm$**16.4**
AOD	Full	$-2.12$	$-{\bf{4.07}}$	$-3.21$	$-1.57$	$-0.709$
		$\pm$2.64	$\pm$**3.40**	$\pm$3.25	$\pm$2.34	$\pm$2.14
	PA	$-{\bf{2.22}}$	$-{\bf{4.49}}$	$-{\bf{2.86}}$	$-{\bf{1.70}}$	$-1.04$
		$\pm$**1.54**	$\pm$**1.55**	$\pm$**2.02**	$\pm$**1.43**	$\pm$1.62
CO${}_{2}$ emissions	Full	$-3.10$	$-6.17$	$-6.43$	$-1.62$	8.35
		$\pm$10.1	$\pm$16.6	$\pm$8.45	$\pm$14.5	$\pm$15.6
	PA	$-2.93$	$-{\bf{5.16}}$	$-4.60$	$-1.35$	9.59
		$\pm$4.82	$\pm$**2.63**	$\pm$4.70	$\pm$11.1	$\pm$14.6

The annual cumulative deforested area for the BLA from the PRODES (Assessment of Deforestation in Brazilian Amazonia) dataset^4^ reports its figures over the period from 1 August to 31 July, with the deforestation year corresponding to the latter year of the record e.g. 2001 data corresponds to the area deforested from 1 August 2000 to 31 July 2001.

Similarly to previous work^38^, we defined the climatological fire period as the five months before the peak in the fire season and six months afterwards. Fire, burned area and AOD all reach their maximum in September, aside from fire in Pará state, which is marginally higher in August. For simplicity, we define September as the peak month across the BLA, thus the climatological fire period is then defined as the period from April-March. This allows us to properly capture the deforestation annual cycle and relate it to the subsequent burning season more directly. All trends and relationships in the following analysis use this framework. The year associated with the annual average is defined relative to the peak month e.g. 2001 represents data from April 2001 to March 2002.

Across the BLA, deforestation rates have decreased substantially over the period from 2001–2016 at a rate of $-1376$ $\pm$ 475 $k{m}^{-2}\ yea{r}^{-1}$ (${R}^{2}$ = 0.73). Deforestation rates rose sharply between 2001–2004, before declining significantly over the subsequent five years. From 2009 onwards, deforestation has been relatively constant from year-to-year, with small increases in 2015 and 2016 compared to the prior stable period.

Active fire counts have declined less sharply over the 2001–2016 period, with decreases of $-1366$ $\pm$ 1628 $yea{r}^{-1}$ (${R}^{2}$ = 0.19) and $-1537$ $\pm$ 1025 $yea{r}^{-1}$ (${R}^{2}$ = 0.42) for the full and precipitation-adjusted time series from 2001–2016, with the latter time series showing a more-significant decline. Trends in burned area have been insignificant from 2001–2015 across the full and precipitation-adjusted time series (0.022 $\pm$ 0.11 and 0.030 $\pm$ 0.068 $yea{r}^{-1}$ respectively).

The trend in AOD has been insignificant from 2001–2016 ($-0.0036$ $\pm$ 0.0045 $yea{r}^{-1}$, ${R}^{2}$ = 0.17) when considering the full time series but is stronger and more significant for the precipitation-adjusted series ($-0.0041$ $\pm$ 0.0029 $yea{r}^{-1}$, ${R}^{2}$ = 0.41).

As would be expected, applying the precipitation-adjustment to the time series typically results in the interannual-variability being dampened, which results in stronger trends as indicated by the narrower 95% confidence intervals and greater ${R}^{2}$ values. Given this the following discussion will refer to only the precipitation-adjusted trends. Values for the full time series are included in Tables 1 and 2 for reference.

Deforestation has declined significantly across all of the major biomass burning states, with ${R}^{2}$ values ranging from 0.62–0.75. Rond${\hat{o}}$nia and Mato Grosso exhibit similar temporal trends to the BLA as a whole, with a sharp rise up-to 2004, followed by a steep decline to 2009 and relative stability thereafter. Pará displays a similar pattern, although the steep decline extends to 2012. Deforestation rates are lower in Rond${\hat{o}}$nia than Mato Grosso and Pará. The dominance of non-forest ecosystems in Tocantins means that deforestation is low overall but the state still sees an initial negative trend that has also been relatively stable from 2009–2016.

Significant declines in fire count are observed in Rond${\hat{o}}$nia, Mato Grosso and Pará with ${R}^{2}$ values of 0.63, 0.56 and 0.35 respectively. The trend in Tocantins is insignificant.

Trends in burned area are negligible and insignificant across the major biomass burning regions.

AOD is generally greatest over Rond${\hat{o}}$nia, where it has declined significantly with an ${R}^{2}$ values of 0.73. Significant negative trends are also observed in Mato Grosso and Pará (${R}^{2}$ values of 0.40 and 0.32 respectively). Trends in Tocantins are small and insignificant.

As shown in Table 2, of the statistically significant relative trends, the declining trend in deforestation across the BLA as a whole is greater than those in precipitation-adjusted fire count and AOD. This is also the case in Rond${\hat{o}}$nia, Mato Grosso, Pará and Tocantins.

### Links between deforestation, fire and aerosol

**Figure 4:**
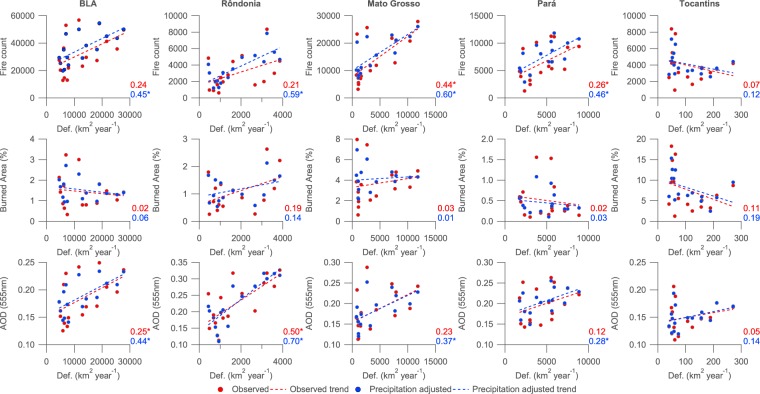
Scatter plots of fire count, burned area and Aerosol Optical Depth (AOD) compared to deforestation rate in the Brazilian Legal Amazon (BLA) and states where significant annual fire occurs (Rond${\hat{o}}$nia, Mato Grosso, Pará and Tocantins). Deforestation rates are from INPE’s PRODES data set. Coefficients of determination (${R}^{2}$) values are included for each sub-figure, where * signifies that the coefficient is significantly different from zero at the 95% confidence level.

Figure 4 illustrates the correlation between deforestation rates and fire count, burned area and AOD across the BLA and the major biomass burning states.

When considering deforestation and fire counts, there is a reasonable degree of correlation across the BLA as a whole (${R}^{2}$ = 0.45). The relationship is stronger in Rond${\hat{o}}$nia (${R}^{2}$ = 0.59) and Mato Grosso (${R}^{2}$ = 0.60), while being insignificant in Tocantins.

There is no significant relationship between burned area and deforestation rate.

Compared to fire count and deforestation, the correlation between deforestation rate and observed AOD time series is reduced, aside from Rond${\hat{o}}$nia where the strongest relationship is observed (${R}^{2}$ = 0.70). Over the BLA as a whole, the correlation is very similar to that between fire count and deforestation, with an ${R}^{2}$ value of 0.44. Over Tocantins, the relationship is insignificant.

Given that the annual cycle of deforestation extends into August and September and would thus coincide with the peak in the burning season, we also performed the correlation analysis by comparing the fire parameters with the deforestation data for the following year e.g. the burning season from April 2001 to March 2002 is compared to the PRODES deforestation annual figures for August 2001 to July 2002. All correlations between fire count, burned area and AOD compared to deforestation are lower for this subsequent analysis, aside from for burned area for Mato Grosso. However, the difference for the burned area for Mato Grosso is minor and insignificant and is likely purely a consequence of random variation given the lack of a linear relationship between it and the deforestation data.

**Figure 5:**
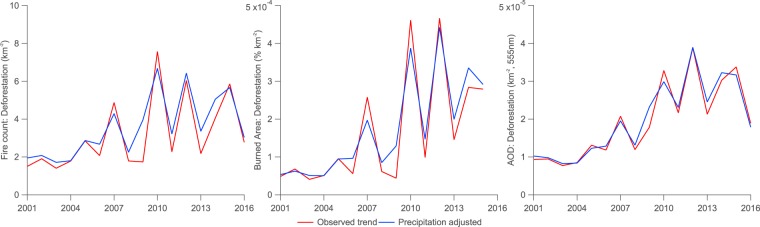
Time series of the ratio of fire count, burned area and Aerosol Optical Depth (AOD) to deforestation rate in the Brazilian Legal Amazon (BLA).

Figure 5 and Table 3 display time series of the ratio of fire count, burned area and AOD to deforestation rate across the BLA for both the observed and precipitation-adjusted time series. The time series can be broadly split into two phases from 2001–2008 and 2009–2016, with all ratios increasing in the later period compared the earlier period. The ratio of fire count to deforestation rate increases by a statistically significant factor of 1.9 $\pm$ 0.6 in the precipitation-adjusted time series. The AOD to deforestation ratio increases by a statistically significant magnitude (factor of 2.3 $\pm$ 0.7 and 2.3 $\pm$ 0.6 for the observed and precipitation-adjusted data respectively), while the change between burned area to deforestation is even larger with a factor of 3.2 $\pm$ 1.6 increase for the precipitation-adjustment time series.

**Table 3:** Comparison between ratios of fire count, burned area, Aerosol Optical Depth (AOD) and total fire-related carbon dioxide emissions to deforestation rate for the Brazilian Legal Amazon across two periods, where Period I is from 2001–2008 and Period II is from 2009–2016, except for burned area which is from 2009–2015. Errors are quoted as 95% confidence intervals based on the standard error for the average ratio in each period. Values in bold are for comparisons that are statistically significant at the 95% confidence level. Ratios are shown for the full period and for the precipitation-adjusted (PA) time series.

Ratio	Data	Period I	Period II
Fire count: Deforestation (# $k{m}^{-2}$)	Full	2.27 $\pm$ 0.79	4.06 $\pm$ 1.51
	PA	**2.45** $\pm$ **0.59**	**4.68** $\pm$ **1.02**
Burned area: Deforestation (% $$k{m}^{-2}$$)	Full	**0.85** $$\times $$ **10**$${}^{-4}$$ $$\pm $$ **0.34** $$\times $$**10**$${}^{-4}$$	**2.54** $$\times $$ **10**$${}^{-4}$$$$\pm $$ **1.25** $$\times $$ **10**$${}^{-4}$$
	PA	**0.86** $\times$ **10**${}^{-4}$ $\pm$ **0.34** $\times$**10**${}^{-4}$	**2.76** $\times$ **10**${}^{-4}$ $\pm$ **0.90** $\times$ **10**${}^{-4}$
AOD: Deforestation	Full ($$k{m}^{-2}$$)	**1.16** $$\times $$ **10**$${}^{-5}$$ $$\pm $$ **0.29** $$\times $$ **10**$${}^{-5}$$	**2.69** $$\times $$ **10**$${}^{-5}$$$$\pm $$ **0.55** $$\times $$ **10**$${}^{-5}$$
	PA	**1.18** $\times$ **10**${}^{-5}$ $\pm$**0.25** $\times$ **10**${}^{-5}$	**2.77** $\times$ **10**${}^{-5}$ $\pm$ **0.47**$\times$ **10**${}^{-5}$
CO${}_{2}$: Deforestation ($Tg\ k{m}^{-2}$)	Full	0.04 $\pm$ 0.02	0.09 $\pm$ 0.04
	PA	**0.04** $\pm$ **0.01**	**0.09** $\pm$ **0.02**

**Figure 6:**
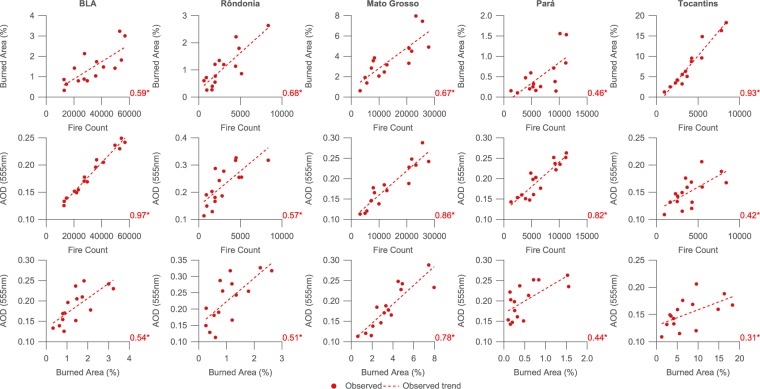
Scatter plots of fire count, burned area and Aerosol Optical Depth (AOD) compared to each other in the Brazilian Legal Amazon (BLA) and states where significant annual fire occurs (Rond${\hat{o}}$nia, Mato Grosso, Pará and Tocantins). Coefficients of determination (${R}^{2}$) values are included for each sub-figure, where * signifies that the coefficient is significantly different from zero at the 95% confidence level.

Figure 6 illustrates the links between fire count, burned area and AOD across the BLA and the major biomass burning states. The precipitation-adjusted data is not considered here as the observed relationship is of primary interest to ascertain how closely linked these parameters are on an annual basis. Burned area and fire count are reasonably correlated over the BLA (${R}^{2}$ = 0.59). Tocantins exhibits the strongest relationship (${R}^{2}$ = 0.93), with the lowest in Pará (${R}^{2}$ = 0.46). AOD and fire count are very closely correlated when examining the BLA as a whole (${R}^{2}$ = 0.97) and over Rond${\hat{o}}$nia, Mato Grosso and Pará, with a weaker correlation over Tocantins. The largest ${R}^{2}$ value is determined when considering the whole Brazilian Amazon, which likely reflects the wider distribution and build-up of smoke across the region. The association between AOD and burned area over the BLA is similar to that of fire count and burned area (${R}^{2}$ = 0.54), while being variable across the region, with the greatest association in Mato Grosso (${R}^{2}$ = 0.78) and lowest in Tocantins (${R}^{2}$ = 0.31).

### Carbon dioxide emissions

**Figure 7:**
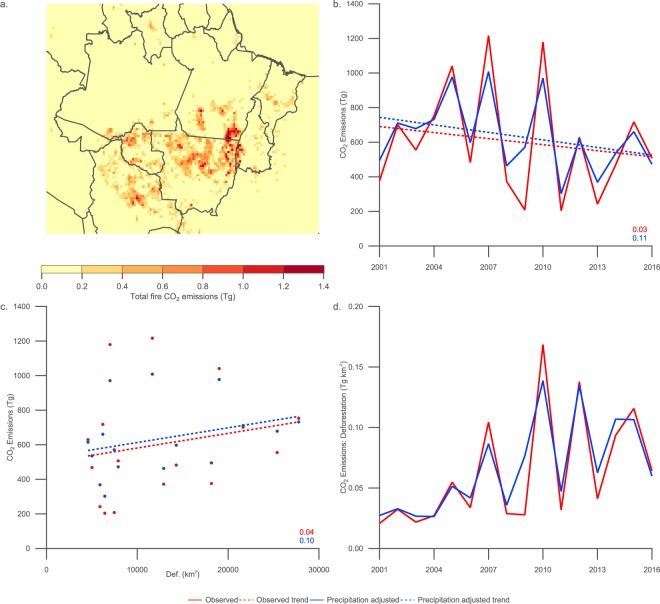
Compilation of figures relating to carbon dioxide emissions from all fire emission classes from 1997–2017. (**a**) Carbon dioxide emissions averaged over the main biomass burning months (August–October) on a 0.25$^{\circ }$ pixel grid. (**b**) Time series and trends in carbon dioxide emissions including coefficients of determination (${R}^{2}$). (**c**) Scatter plot of carbon dioxide emissions compared to deforestation rate including coefficients of determination (${R}^{2}$). (**d**) Ratio of carbon dioxide emissions to deforestation rate.

Figure 7a displays the spatial distribution of fire-related carbon dioxide emissions from the Global Fire Emissions Database (GFED4)^39^. Given that the emissions are based on remote-sensing fire and burned area observations, the spatial distribution of carbon dioxide emissions closely reflects the fire and burned area distributions in Fig. 1. The overlap between the number of fires and extent of the area burnt in north-eastern Mato Grosso on the border with Tocantins results in substantial carbon dioxide emissions from deforestation and forest degradation fires, along with savannah fires. Significant carbon dioxide emissions occur in Rond${\hat{o}}$nia and northern Mato Grosso due to deforestation and degradation fires. Based on the GFED4 emissions categorisation, agricultural waste burning makes a small contribution to the total fire-related emissions, with a maximum over central Mato Grosso.

The annual cycle in fire-related carbon dioxide emissions is included in Fig. 2, showing that emissions peak across the BLA from August-October, reflecting the underlying trends in fire count and burned area. Emissions peak in September across all states, aside from Pará, which is consistent with its fire and burned area annual profile. Significant emissions occur in Mato Grosso during July as well, contributing 54% of the total emissions across the BLA.

Figure 7b and Table 1 illustrate that fire-related carbon dioxide emissions have seen minor and insignificant absolute declines of $-11.6$ $\pm$ 38.0 $Tg$ $yea{r}^{-1}$ (${R}^{2}$ = 0.03) and $-14.5$ $\pm$ 23.9 $Tg$ $yea{r}^{-1}$ (${R}^{2}$ = 0.11) from 2001–2016 in the observed and precipitation-adjusted time series respectively. Relative changes in emissions are all smaller than those for deforestation and are shown in Table 2. The negative trend in precipitation-adjusted emissions in Rond${\hat{o}}$nia is the only statistically significant trend within the analysis. These trends are consistent with the combination of the declines in fire count and burned area. As a consequence, the relationship between carbon dioxide emissions and deforestation rate across the BLA shown in Fig. 7c is insignificant, with ${R}^{2}$ values of 0.04 and 0.10 for the standard and precipitation-adjusted emissions respectively. As would be expected, the relationship between carbon dioxide emissions and fire counts, burned area and AOD is very strong and statistically significant, with ${R}^{2}$ values of 0.89, 0.83 and 0.84 respectively over the BLA.

The ratio between fire-related carbon dioxide emissions and deforestation rate is shown in Fig. 7d. Given the trends in deforestation and carbon dioxide emissions shown previously, the ratio increases substantially over the period from 2001–2016 with a statistically significant factor of 2.2 $\pm$ 1.0 increase when comparing the period from 2009–2016 with that from 2001–2008 for the precipitation-adjusted emissions.

## Discussion

The spatial distribution of deforestation and fire count tend to overlap each other across the BLA as shown in Fig. 1, although there are some divergences e.g. significant deforestation has occurred in central Pará but without clear enhancements in fire count as well as burned area. Grassland and shrubland fires dominate in the east where deforestation is more limited. Burned area shows the greatest magnitudes in the east rather than in the main deforestation areas of Rond${\hat{o}}$nia, central Mato Grosso and south-western Pará. AOD is greatest over Rond${\hat{o}}$nia and western Mato Grosso, reflecting the spatial distribution of fire and burned area but with a shifted maxima due to the predominant easterly air flow across the region. The spatial distribution of fire-related carbon dioxide emissions shown in Fig. 7a is closely coupled to the fire and burned area distribution, with the greatest emissions in south-eastern Pará and the border between Mato Grosso and Tocantins, illustrating the importance of burned area to the total emissions.

Similarly to prior studies^5,38^, we see a clear peak in fire, burned area, AOD and fire-related carbon dioxide emissions in August/September during the dry season. This is consistent with the fact that the main deforestation timing occurs in May-July, while fire is more prevalent towards the end of the dry season.

As illustrated in Fig. 3 plus Tables 1 and 2, deforestation rates declined significantly from 2001–2016 across the BLA and the major biomass burning regions, while negative trends in fire count, burned area and AOD have been either less significant or negligible in comparison. However, the observed trends in fire count and AOD in particular are strongly modulated by inter-annual variability in precipitation based on comparing the observed and precipitation-adjusted trends. When accounting for inter-annual variability in precipitation, the declining trends in fire count and AOD are statistically significant across the BLA and the major biomass burning states aside from in Tocantins. In Rond${\hat{o}}$nia, the declines in fire count and AOD occur at comparable relative rates to deforestation. In Mato Grosso and Pará, the relative decline in fire count is also comparable to the relative decline in deforestation. Direct comparisons between the annual averages in Fig. 4 further illustrate these findings, with strong relationships between fire and deforestation in Rond${\hat{o}}$nia and Mato Grosso and between AOD and deforestation in Rond${\hat{o}}$nia. We find a weaker association between AOD and deforestation than a prior recent study^19^, although they considered a different spatial area, a shorter time period from 2001–2012 and accounted for inter-annual variability by simply excluding drought-years; applying the same analysis across our study period over the BLA yields smaller correlation coefficients than their analysis, which is likely a consequence of the changing relationship between the parameters from 2013–2016 compared to 2001–2012.

Trends in burned area are insignificant over our study domain and time period in both the observed and precipitation-adjusted time series, aside from Tocantins where a small increase is observed in the precipitation-adjusted data. Previous work^38^ has also observed a positive trend from 1998–2015 in the east of the BLA, with a small negative trend in the west. Similar to our results but over South America as a whole, the overall trends they observed were statistically insignificant. Compared to the GFED3 iteration of the burned area product, the GFED4 iteration used here includes small fires from pixels that the active fire algorithm indicated as fire occurrences that were not flagged by the burned area algorithm. This substantially increased the burned area due to small fires, particularly in regions dominated by fires of less than 500 $m$^40–42^. The negative trend in fire count and AOD, but insignificant trend in burned area (Figs 3 and 6) suggests a shift in fire dynamics and practices across the period. One possible mechanism could be individual fires burning a larger area with lower fuel load that would be consistent with a transition towards fewer deforestation fires and more agricultural, grassland and forest degradation fires.

Fire-related carbon dioxide emissions illustrated in Fig. 7b plus Tables 1 and 2 show minor or insignificant declines across the period from 2001–2016. This is consistent with the trends in fire and burned area, which form the basis of the emissions estimate. The relationship between carbon dioxide emissions and deforestation (see Fig. 7c) is very weak regardless of whether interannual variability in precipitation is considered or not. Fire-related carbon dioxide emissions are strongly correlated with AOD (${R}^{2}$ value of 0.84 for the BLA as a whole). This association plus the close coupling between AOD and fire count (${R}^{2}$ value of 0.97 for the BLA as a whole), and to a lesser extent burned area, illustrates the tight control of fire on the magnitude of the aerosol burden and implies that AOD is potentially a useful measurement diagnostic to represent total emissions from fire in the BLA.

The ratios of fire count, burned area, AOD and fire-related carbon dioxide emissions to deforestation rate shown in Figs 5 and 7d and reported in Table 3 demonstrate a clear de-coupling between these parameters and deforestation over the period from 2001–2016. When splitting our study period into phases from 2001–2008 and 2009–2016, we observe statistically significant differences of factors of approximately 2–3 when inter-annual variability is considered. The large inter-annual variations in the ratios themselves point to factors other than deforestation playing a major role in regulating fire parameters, AOD and fire-related carbon dioxide emissions. Our analysis confirms and expands on prior work examining the number of fire counts per area of deforestation^7,27^.

A complicating factor in any analysis using the PRODES deforestation dataset is whether it accurately reflects the scale of forest loss over the study period. Prior work^43^ has suggested Brazil’s deforesters were avoiding detection based on comparisons with the University of Maryland Global Forest Change (UMD GFC) dataset^44^, which identified a divergence between the two forest loss estimates after 2008 when the Brazilian government began using PRODES as part of enforcement activities. Furthermore, they identified spatial biases between the datasets in northern Mato Grosso and north-eastern Pará, which they postulate are driven by landowners having the greatest incentives and knowledge to avoid detection. Subsequent analysis^2^ has also compared PRODES to the UMD GFC, as well as estimates from high-resolution Landsat satellite imagery^45^, finding that both methods underestimated forest loss, with a larger divergence from 2008–2013. However, when comparing PRODES with human clearing of primary forest in the UMD GFC, very good agreement in terms of the substantial negative trend and area of deforestation from 2001–2013 has been found, without any apparent discrepancy after 2008^2,45^. When comparing the different datasets, the annual estimates were found to differ more significantly, with PRODES overestimating deforestation by up to 25% and underestimating by up to 18% compared to UMD GFC, with no clear pattern of PRODES underestimating deforestation. The minimum mapping unit of 6.25 $ha$ for PRODES has been identified as a contributing factor to PRODES missing small clearings^2,43^, with one study^43^ estimating this contributed an average of 5,000–6,000 $k{m}^{2}$ of additional forest loss on an annual basis. Areas identified as previously deforested are not monitored under PRODES, which has been estimated to add a further 2,500 $k{m}^{2}$ of forest clearing annually^43^. By 2013, non-primary forest clearing and primary forest degradation (53%) has been estimated to contribute a comparable area to human clearing of primary forests (47%)^2^. Based on the literature cited above and in the introduction, it is clear that PRODES underestimates total forest loss in the BLA, although the deforestation figures themselves appear robust and consistent with other studies^2,45^.

Our results clearly demonstrate a de-coupling between fire and associated emissions of particulate and gas-phase material from deforestation of the Brazilian Amazon. This is consistent with prior studies^7,13,27–29^ illustrating the increasing role of non-deforestation forest loss that has been linked to degradation of the forest ecosystem and drought-related fires. This suggests that the non-deforestation related drivers of fire in the region are highly pertinent when considering controls on the aerosol burden as well and thus have implications for weather, climate, human health and ecosystems across the BLA and the wider region. Forest loss from deforestation reported from PRODES forms the basis for official greenhouse gas emissions produced by the Brazilian government; our results reinforce the conclusion of prior studies^2,7,43^ that failure to include the significant contribution from non-deforestation related drivers of forest loss and fire will result in such estimates being biased low, both now and in the future. This has major implications for Brazil’s greenhouse gas emission estimates and associated climate change mitigation policies. Future studies should look to quantify the contribution of different forest loss pathways to aerosol and greenhouse gas emissions, including black carbon and methane respectively, and their resultant impacts.

Dry-season length over the southern BLA has increased over recent decades^46,47^, increasing the risk and impact of fire in the region. Climate-driven changes that promote increased fire risk are expected to continue during the 21st Century^3,48,49^. Furthermore, areas in the southern and eastern BLA where deforestation is most active (in both the present and future scenarios) are at the highest risk of drought^50^. Global Earth system models predict that deforestation itself is expected to lead to a warmer and drier climate in the BLA^51–55^. These climate and deforestation-driven influences may therefore combine to increase fire susceptibility in the region, with potential feedbacks reinforcing the changes in fire dynamics and forest degradation discussed previously. Such changes will also influence the concentration, spatial distribution and impacts of the aerosol burden and carbon dioxide emissions, which should be explored in future studies.

Estimates of deforestation rate from PRODES for 2018 indicate a continuation of the trend towards increased deforestation since 2014, with a sharp increase in fire counts in 2018 as well. Our results build on prior studies and suggest that should this level of deforestation be maintained or increased, then the fire susceptibility and degradation of the Amazon will be exacerbated and have consequences for aerosol and greenhouse gas emissions. This is particularly important since the Paris Agreement Nationally Determined Contributions from Brazil is explicit on zero illegal deforestation by 2025 and to reforest 12 million hectares by 2030. The new Brazilian government that took office in January 2019 is dominated by the agro-business, which is the sector pressuring for more Amazonian deforestation and conversion to pasture and farmland. Since these actions could have a strong negative impact on atmospheric carbon dioxide concentrations, as well as large emissions of black carbon and methane, policies should consider the exacerbating factors identified in our study and their consequences.

## Data and Methods

### Datasets

The Tropical Rainfall Measuring Missions (TRMM) Multisatellite Precipitation Analysis (TMPA) provides combined precipitation estimates from multiple satellites and gauge analysis where possible on a 0.25$^{\circ }$ by 0.25$^{\circ }$ grid at three-hourly intervals^56^. We use the monthly rainfall data product (TMPA/3B43 Rainfall Estimate L3 1 month 0.25$^{\circ }$ $\times$ 0.25$^{\circ }$ V7) retrieved from the GIOVANNI (online data system http://disc.sci.gsfc.nasa.gov/giovanni), which is developed and maintained by the NASA Goddard Earth Sciences Data and Information Services Center (GES DISC, last accessed 23 May 2018, 10.5067/TRMM/TMPA/MONTH/7).

The Instituto Nacional de Pesquisas Espaciais (INPE) provides annual cumulative deforested area for the BLA via the PRODES (Assessment of Deforestation in Brazilian Amazonia) dataset^4^. Only deforestation of primary forest is mapped, with clearing of secondary forest regrowth not considered. The minimum mapping unit for images is 6.25 $ha$, which results in smaller clearings being omitted. The annual figures are reported from 1 August to 31 July, with the deforestation year corresponding to the latter year of the record e.g. 2016 data corresponds to the area deforested from 1 August 2015 to 31 July 2016. INPE also provides the DETER (Detection of Deforested Areas in Real Time) dataset^57^, which reports real time quick look estimates of deforestation and cumulative monthly deforested area for the BLA. The PRODES dataset is provided by INPE via their website http://www.obt.inpe.br/OBT/assuntos/programas/amazonia/prodes (last accessed 23 May 2018). Similarly, the DETER dataset is also provided by INPE via their website http://www.obt.inpe.br/deter/dados/ (last accessed 4 June 2018).

The Moderate Resolution Imaging Spectroradiometer (MODIS) on the Terra satellite platform provides active fire data over the region. Maps of average fire count on a 0.25$^{\circ }$ pixel grid were generated. Only fire counts assigned a confidence class of high (80% $\le$ confidence $\le$ 100%) are included. The active fire data is released under the MODIS Collection 6 algorithm^58^ from the Fire Information for Resource Management System (FIRMS). The MODIS Collection 6 algorithm demonstrates improved performance compared to prior versions with respect to translucent and opaque clouds, as well as the omission of large fires obscured by thick smoke. The data is produced by the University of Maryland and was acquired from the online Fire Information for Resource Management System (FIRMS https://earthdata.nasa.gov/earth-observation-data/near-real-time/firms; specific product: MCD14ML, accessed 4 June 2018).

The fourth version of the Global Fire Emissions Database (GFED4) provides monthly burned area at 0.25$^{\circ }$ spatial resolution^41^. The data combines MODIS Collection 6 burned area maps with active fire data from the TRMM Visible and Infrared Scanner (VIRS) and the Along-Track Scanning Radiometer (ATSR) family of sensors. The data was retrieved via the GFED website https://www.globalfiredata.org/data.html (last accessed 23 May 2018).

The Multiangle Imaging Spectroradiometer (MISR)^59–61^ instrument onboard the Terra satellite platform measures Aerosol Optical Depth (AOD), and has been found to agree well with MODIS AOD^62^. We use the level 3 component global aerosol product data at a 0.50$^{\circ }$ pixel resolution, which was retrieved from the NASA Atmospheric Science Data Center (ASDC https://eosweb.larc.nasa.gov/; specific product: MIL3MAEN, last accessed 23 May 2018).

Monthly fire-related carbon dioxide emissions are from GFED4^39–41^, which boosted small fire burned area compared to prior iterations. The data is at a 0.25$^{\circ }$ spatial resolution and was retrieved via the GFED website https://www.globalfiredata.org/data.html (last accessed 31 October 2018). The database reports a break-down of fire-related emissions from different fire classes, including tropical forest fires (including both deforestation and degradation), combined savannah, grassland and shrubland fires, as well as agricultural waste burning. GFED4 uses the MODIS collection 6 burned area data referred to above, alongside fuel mass and emission factors; as a result we would expect similar responses between the GFED4 emissions and burned area observations used in our study. Uncertainties in regional carbon emissions from GFED4 are driven by a number of factors such as the importance of small (undetected) fires in a region, burned area and emission factors^39^; formal estimates of the uncertainty are not provided for GFED4 due to difficulties in assessing the uncertainties in these and other factors. A best-guess uncertainty of 50% at the 1$\sigma$ level at regional scales is provided^39^.

Data masks for the BLA and the states within it (Acre, Amazonas, Rond${\hat{o}}$nia, Roraima, Mato Grosso, Pará, Amapá, Tocantins and Maranhão) were calculated on 0.25$^{\circ }$ and 0.50$^{\circ }$ pixel grids to match the corresponding dataset. These masks approximate the geographical limits of the BLA and state borders.

Data from MODIS and MISR from November 2000 to May 2017 is used in the analysis, while GFED4 data is from November 2000 to July 2016. Deforestation data from PRODES covers the entire study period, while the DETER dataset runs from May 2004 to May 2017. TRMM data is used from November 1998 (two-years before the other datasets) to May 2017.

### Precipitation-adjusted annual trend method

Given the climate related drivers of fire in the BLA, we performed a statistical analysis of the fire and aerosol satellite data to remove the influence of precipitation-induced interannual-variability on the trends across the BLA. We followed a method outlined in a prior study^63,64^ and its adaptation in a subsequent study^38^.

The method defines a linear model to estimate the impact of precipitation-induced changes in fire and aerosol parameters, which is optimised for each individual grid cell so as to prevent over fitting. Fire and burned area were analysed alongside TRMM data on a 0.25$^{\circ }$ grid, while the aerosol-related parameters were analysed on a 0.50$^{\circ }$ with the TRMM data averaged-up to match. Unlike the prior study that our method is based on^38^, we defined a single month for the annual peak for all grid cells to simplify the analysis and better represent the state and region-wide scales that are of primary interest to our analysis. Based on the annual cycles in fire, burned aerosol and AOD, September is defined as the peak month of the biomass burning season. All parameters reach their maximum in September, aside from fire in Pará state, which is marginally higher in August. The burning season is then defined as the period from April-March i.e. five months before the peak and six months afterwards.

The antecedent precipitation ($AP$) is defined as the mean monthly precipitation ($P$) prior to the peak in the biomass burning season ($m$) over a period of 1 to 24 months ($T$). This results in 24 separate annual time series for each grid cell ($x,y,t$) that are combined with the annual time series of fire and aerosol to explore their relationship:

1 $$A{P}_{x,y,t}=\frac{{\sum }_{i=m-{T}_{x,y}}^{i=m-1}\ {P}_{x,y,i}}{{T}_{x,y}}$$

Time series of $AP$ and fire, burned area, AOD and carbon dioxide emissions (referred to collectively as fire parameter ($FP$) below) are de-trended via linear regression in Eqs 2 and 3 respectively:

2 $$AP=A{P}_{IAV}+A{P}_{TREND}$$

3 $$FP=F{P}_{IAV}+F{P}_{TREND}$$

$A{P}_{TREND}$ and $F{P}_{TREND}$ are thus trend-corrected anomalies after the removal of inter-annual variability (IAV).

Inter-annual variability in fire and aerosol is likely strongly controlled by precipitation and is explored in Eq. 4, where their time series are used to optimise a no-intercept linear regression for each of the 24 $A{P}_{IAV}$ time series. The equation minimises the sum of the least square errors (${\varepsilon }_{x,y,t}$) through the optimally-fitted parameter, $b{0}_{x,y}$:

4 $$F{P}_{IA{V}_{x,y,t}}=b{0}_{x,y}\times A{P}_{IA{V}_{x,y,t}}+{\varepsilon }_{x,y,t}$$

The correlation between the time series of $F{P}_{IA{V}_{x,y,t}}$ and $A{P}_{IAV}$ is then calculated to determine the strongest relationship between precipitation-driven variability and trends in fire and aerosol. This provides the most appropriate time span (in months) to represent the antecedent precipitation for a given grid box and fire parameter.

The precipitation-induced time series, $F{P}_{precipitation}$, is then determined via Eq. 5 as a function of the antecedent precipitation for each grid box for each year ($A{P}_{x,y,t}$):

5 $$F{P}_{precipitation}=b{0}_{x,y}\times A{P}_{x,y,t}$$

Finally, the precipitation-adjusted time series ($F{P}_{PA}$) can be determined via Eq. 6 as the difference between the observed and precipitation-induced time series:

6 $$F{P}_{PA}=F{P}_{IAV}+F{P}_{trend}-F{P}_{precipitation}=FP-F{P}_{precipitation}$$

This provides annually averaged precipitation-adjusted parameters across the BLA, which can be analysed and compared with the observations.

An important factor for the precipitation-adjustment analysis is that the onset of the dry and wet seasons varies spatially across the BLA, as well as inter-annually. For illustrative purposes, we examined the month where average rainfall was above and below 100 $mm$ for two consecutive months to evaluate the transition from the wet-to-dry season. The transition between seasons is typically sharp, especially when considering monthly-averages, with 100 $mm$ being used as a broad indicator in previous studies^65^. Over the period used in our analysis from November 1998 to May 2017, the BLA as a whole typically transitions to the wet season in October in 79% of our annual samples, with the transition occurring in September and November in 11% of the samples each. Over Rond${\hat{o}}$nia the typical month is October (68%), followed by November (21%) and September (11%). The transition occurs in October over Mato Grosso consistently (95% of samples) with the remainder in November. The transition month is more variable over Pará, with 11% of instances occurring in September, 47% in October, 21% in November, 16% in December and 5% in January; Pará is more sensitive to the choice of cutoff value due to it being close to its average rainfall from September-November. Increasing the cutoff value to 150 $mm$ provides a clearer picture with 55% of the samples transitioning in December, followed by 25% in January and 15% in November. Tocantins is evenly split between October (53%) and November (47%).

While the spatial differences across the BLA are captured by the precipitation-adjustment as it considers trends at individual grid cells, areas with greater interannual-variability in the transitions between the wet and the dry season will introduce some uncertainty into the choice of time span for the antecedent precipitation. However, the uncertainty in the time span is likely small, especially given that the typical transition month differs by at most a month in the examples above.

## Data Availability

All data used in the manuscript is available publicly. Further details are given in the methods section and the relevant references.
